# Authors’ Response to Peer Reviews of “Evaluating Population Density as a Parameter for Optimizing COVID-19 Testing: Statistical Analysis”

**DOI:** 10.2196/27258

**Published:** 2021-02-03

**Authors:** Karim I Budhwani, Henna Budhwani, Ben Podbielski

**Affiliations:** 1 CerFlux, Inc Birmingham, AL United States; 2 University of Alabama at Birmingham Birmingham, AL United States; 3 Protective Life Corp Birmingham, AL United States

**Keywords:** infectious diseases, testing, per capita, population density, policy, coronavirus, SARS-CoV-2, COVID-19

*Authors' response to peer reviews for “Evaluating Population Density as a Parameter for Optimizing COVID-19 Testing: Statistical Analysis.”*

## Response to Round 1 Reviews

### Reviewer: Anonymous

#### General Comments

Dear anonymous reviewer [1], we would like to begin by conveying to you our deep appreciation for your assistance in refining this short paper [2] so that it is suitable for broader consumption. It is our aspiration that this paper will contribute positively to advancing knowledge in this domain. We have fully addressed all your recommendations and are pleased to submit a revised manuscript. Thank you for your expert assistance in this endeavor.

#### Specific Comments

##### Major Comments

You raise excellent points. We are happy to note that some of these points are a result of automatically transferring our manuscript from the preprint server. We submitted our manuscript originally to a preprint server with the goal of sharing our analysis and viewpoint in a timely and nonintimidating manner by way of a short report. The title, format, and manuscript text were rapidly copied from the general preprint server edition during the automatic transfer process.

The revised manuscript addresses the following:

1. The title has been updated to “Evaluating Population Density as a Parameter for Optimizing COVID-19 Testing: Statistical Analysis.”
2. Absolute terms from the preprint report have been modified.
3. The elevator vs football field “visual” expression was included deliberately in the original report as a means to make the role of density in SARS-CoV-2 viral transmission readily apparent to a broad audience. In order to address your concern, we have removed a reference to this expression in the *Results* section; however, in keeping with the original intent of reaching a broader audience, we would prefer to retain the expression in the *Introduction*.
4. We have included statements on limitations. Thank you for noting this gap.
5. We agree that a cost-effectiveness analysis is warranted after feasibility and acceptability have been established, but due in part to the word limit for short papers, we are unable to explore these differences. We believe that a paper on the costs and financial consequences of different testing strategies is warranted, potentially in follow-up analyses. Thank you for this recommendation.
6. In response to whether or not adjusted testing strategies based on population density (or similar measures) have been successfully done elsewhere: population density–based testing is novel, having (to our knowledge) only been employed in HIV research through network tracing in urban metropolitan areas. This gap in knowledge in terms of the benefit of population density testing is likely because we have not encountered many agents that are as infectious and persistent as SARS-CoV-2. This short paper is an initial step to illustrate to the scientific community that targeted approaches may be warranted when community spread occurs through close contact that is more likely in tightly packed communities.

### Reviewer: AAA

#### General Comments

Dear reviewer AAA [3], we would like to begin by conveying to you our deep appreciation for your assistance in refining this short paper so that it is suitable for broader consumption. It is our aspiration that this paper will contribute positively to advancing knowledge in this domain. We have fully addressed all your recommendations and are pleased to submit a revised manuscript. Thank you for your expert assistance in this endeavor.

#### Specific Comments

##### Minor Comments

1. Your recommended heading change has been made in the revised manuscript.
